# Less Than Desired: Sexual Expression and Subsequent Loneliness Among Older Married Couples

**DOI:** 10.1093/geroni/igaf122.841

**Published:** 2025-12-31

**Authors:** David Warner, Heidi Lyons

**Affiliations:** The University of Alabama at Birmingham, Birmingham, Alabama, United States; Oakland University, Rochester, Michigan, United States

## Abstract

Older married couples continue to engage in sexual activity—even as the frequency wanes with age—and this is associated with better physical and mental health. Sexual activity, however, is but one component of sexual expression. Indeed, we have previously shown there is considerable heterogeneity in sexual expression among older couples when behavior, desire, and attitudes are considered simultaneously, and objectively low levels of sexual activity may or may not be congruent with expectations. Some couples, for example, with infrequent behavior and ambivalent about whether sex is an important part of life still indicate that they are having sex less than desired. The implications of this incongruence for subsequent well-being are unclear. We address this gap with data on 595 heterosexual couples in long-term marriages from Rounds 2 and 3 of the National Social Life, Health, and Aging Project (NSHAP). Using latent class analysis with distal outcomes, we examined how couple-level sexual expression predicted subsequent loneliness—an indicator of unmet psychosocial need—for both spouses. We found that latent classes characterized by less than desired sexual activity (not simply low activity) were associated with higher subsequent loneliness among both wives and husbands, and this effect was strongest for those classes where couples viewed sex as important part of life. This pattern was robust to controls for physical health and marital quality. The findings add nuance to our understanding of the health consequences of sexual activity and point to the importance of evaluating behaviors in the context of expectations.

